# Anterior Column Reconstruction Through a Posterior Corridor in the Lower Thoracic Spine and Thoracolumbar Junction: Early Radiographic Outcomes Using a Conformable Mesh Interbody Implant

**DOI:** 10.3390/jcm15208077

**Published:** 2026-10-17

**Authors:** Phil Bohlen, Malia McAvoy, Nguyen Tran, Nicholas Dietzman, Malek Bashti, Jan Bredow, Peer Eysel, Mahesh Karandikar, Woo-Keun Kwon, Christoph P. Hofstetter

**Affiliations:** 1 Department of Neurological Surgery, University of Washington, Seattle, WA 98104, USA; 2 Department of Orthopedics and Trauma Surgery, Faculty of Medicine, University Hospital Cologne, University of Cologne, 50937 Cologne, Germany; 3 Department of Orthopedics and Trauma Surgery, Krankenhaus Porz am Rhein, University of Cologne, 51149 Cologne, Germany; 4 Department of Neurosurgery, Korea University Guro Hospital, Korea University College of Medicine, Seoul 08308, Republic of Korea

**Keywords:** thoracic spine, thoracolumbar spine, anterior column reconstruction, segmental kyphosis, interbody fusion, radiographic outcomes

## Abstract

**Background/Objectives:** Restoration of anterior column support in the lower thoracic spine and thoracolumbar junction can be challenging in the setting of segmental kyphosis, particularly when endplate integrity is compromised. A conformable mesh interbody implant can be introduced in a collapsed configuration through a transforaminal corridor and expanded in situ to accommodate the geometry of the prepared intervertebral space. This study evaluated early radiographic outcomes following anterior column reconstruction with a conformable mesh interbody implant. **Methods:** This retrospective single-centre study was conducted with 12 patients who underwent thoracic or thoracolumbar anterior column reconstruction using a conformable mesh interbody implant between April 2024 and March 2026. The heterogeneous cohort included degenerative disease, trauma, infection, hardware failure and pseudarthrosis. Segmental Cobb, anterior disc height and posterior disc height were measured preoperatively, immediately postoperatively and at 3-month follow-up. Paired radiographic changes were analyzed using the Wilcoxon signed-rank test. **Results:** Median segmental Cobb angle improved significantly from −16.2° preoperatively to −7.9° immediately postoperatively (median change +6.4°, *p* = 0.003), with correction maintained at 3 months (+5.5° versus baseline, *p* = 0.008). Anterior disc height increased from 6.0 to 9.2 mm immediately postoperatively (*p* = 0.004) and remained significantly greater than the baseline at 3 months despite a small early reduction in anterior disc height (+2.3 mm, *p* = 0.013). Posterior disc height increased from 4.3 to 7.6 mm (*p* = 0.021) and remained improved at 3 months (+2.1 mm, *p* = 0.014). **Conclusions:** Anterior column reconstruction through a posterior surgical corridor using a conformable mesh interbody implant appeared feasible in the lower thoracic spine and thoracolumbar junction. Significant restoration of segmental alignment and disc height was achieved, with most radiographic correction maintained at 3 months. These preliminary findings support the short-term maintenance of radiographic correction achieved with this reconstructive strategy.

## 1. Introduction

The reconstruction of the anterior column is an important component of surgical treatment especially in selected thoracic and thoracolumbar pathologies [1]. Loss of anterior column height and subsequent kyphotic deformity may occur in the setting of traumatic injury, disc space collapse, infectious disease, or pseudarthrosis or after extensive decompression following an initial surgery. If not adequately addressed, insufficient anterior support may contribute to segmental kyphosis, loss of correction postoperatively or increased loading of the posterior instrumentation with mechanical failure over time [1,2,3]. Accordingly, restoration of disc space height and sagittal alignment is a central objective of reconstructive surgery, as the anterior column contributes to not only indirect neural decompression but also load sharing between the anterior and posterior spinal columns [2].

While certain kyphotic deformities benefit from column reconstruction for optimal outcome, it can be technically demanding. Traditional anterior or lateral approaches provide direct access to the vertebral body and disc space but also require substantial exposure and can be associated with increased morbidity in spine surgery [4,5]. To avoid this, posterior and posterolateral approaches can be performed without additional transthoracic or retroperitoneal exposure and to allow decompression, instrumentation and deformity correction. However, the insertion of a rigid interbody implant through a constrained posterior corridor may be limited because of anatomical constraints. In particular, when anterior column collapse is accompanied by segmental kyphosis, restoration of sagittal alignment often requires a lordotic cage with a larger footprint and greater angulation. Introducing such an implant through a small posterior working corridor is technically demanding and may, in certain circumstances, increase the procedural risk. Therefore, implant insertion itself frequently represents the limiting step of posterior addressed pathologies of the anterior column [5,6]. This challenge is particularly pronounced in the thoracic spine, where the spinal cord, narrow pedicles, rib heads and the thoracic cage restrict the available working corridor. Spine surgeons face these difficulties further in revision surgery, infectious disease, fractures and multilevel reconstructions, in which distorted anatomy or pre-existing instrumentation may complicate the insertion of a conventional rigid interbody implant [7]. These complex pathologies may also be associated with compromised or eroded vertebral endplates, resulting in irregular intervertebral geometry and limiting stable apposition of a fixed-geometry cage. In this setting, reconstruction must address both the restricted posterior working corridor and the challenge of achieving adequate implant–endplate contact without excessive focal loading.

Conformable interbody implants offer a reconstructive strategy that may address both of these limitations. Rather than inserting a rigid implant at its final dimensions, the mesh is introduced through a small corridor, and after positioning, it is progressively filled with bone graft in situ, which allows expansion within the prepared interbody space [8,9,10].

The pivotal SCOUT (Spineology Clinical Outcomes Trial) trial for a conformable interbody cage demonstrated favourable outcomes, including pain reduction, sufficient fusion rates and a good safety profile with no serious implant-related adverse events in 102 patients. However, this study was conducted in a relatively homogenous cohort, comprising primarily patients with single-level lumbar degenerative disease [8]. In clinical practice, the conformable mesh cage technology may also offer advantages in scenarios with increased complexity, such as multilevel collapsed disc spaces, fractures, infectious disease as well as revision surgery, where anatomical distortion renders standard cage insertion difficult [11,12,13]. The evidence published so far leaves a significant gap in these scenarios describing the broader applicability of the proposed method.

The purpose of this study was therefore to evaluate whether anterior column reconstruction using an in situ expanded conformable mesh interbody implant can be achieved through a posterior corridor in thoracic and thoracolumbar spine surgery. We specifically investigated early radiographic outcomes of sagittal alignment and disc height following reconstruction in a heterogeneous cohort, including degenerative disease, fractures, infection and revision surgery.

## 2. Materials and Methods

### 2.1. Study Design and Patient Selection

This retrospective single-centre study included patients who underwent thoracic or thoracolumbar reconstruction using a conformable mesh interbody implant (OptiMesh^®^, Spineology Inc., St. Paul, MN, USA) between April 2024 and March 2026. The patients were identified from an institutional operative database. The study was approved by the institutional review board of the University of Washington (Protocol #07742).

Patients were eligible for inclusion if a conformable mesh interbody implant was inserted at a thoracic or thoracolumbar level and if both preoperative and postoperative radiographic imaging were available for review. Patients treated exclusively at lumbar levels were excluded. The final study cohort comprised 12 patients, with implant levels ranging from T8-T9 to T12-L2. The cohort included both index and revision surgeries and encompassed traumatic, degenerative, disc-related, infectious disease, mechanical failure and pseudarthrosis. Given this heterogeneity and limited sample size, the patients were analyzed as a single radiographic cohort without disease-specific subgroup comparisons.

### 2.2. Data Collection

Demographic and perioperative variables were obtained from the medical record and institutional operative documentation. Collected variables included the following: age, sex, Body Mass Index (BMI), American Society of Anesthesiologists-Class (ASA) classification, primary pathology, anatomical region, revision status, intervention type, operative duration, estimated blood loss and hospital length of stay. Revision was defined as reconstruction performed at a previously operated segment or modification of a pre-existing instrumented construct.

### 2.3. Surgical Technique

All procedures were performed using a posterior or posterolateral approach, including open, minimally invasive and endoscopic posterior techniques.

Following exposure and neural decompression as indicated, the target reconstructive interval was prepared through a transforaminal or posterolateral corridor. If necessary, disc material and cartilaginous endplate tissue were carefully removed while preserving the structural integrity of the endplates. A conformable mesh interbody implant was then inserted in its unfilled configuration and progressively packed in situ with ready-to-use demineralized allograft until adequate disc space reconstruction and anterior column support were achieved. Posterior segmental instrumentation was placed or revised according to the requirements of the individual case. Additional corrective maneuvers, including patient positioning, decompression, facetectomy, rod contouring and posterior instrumentation were performed as clinically indicated and were hence not standardized across the cohort. Accordingly, changes in segmental alignment reflect the overall reconstructive procedure and cannot be solely attributed to the expansion of the conformable mesh implant.

### 2.4. Radiographic Assessment

Radiographic measurements were obtained from available sagittal radiographs, computed tomography scans or magnetic resonance imaging, as appropriate, at the following three time points:

1. Preoperative;
2. Immediate postoperative;
3. Three months postoperative.

**Figure 1:**
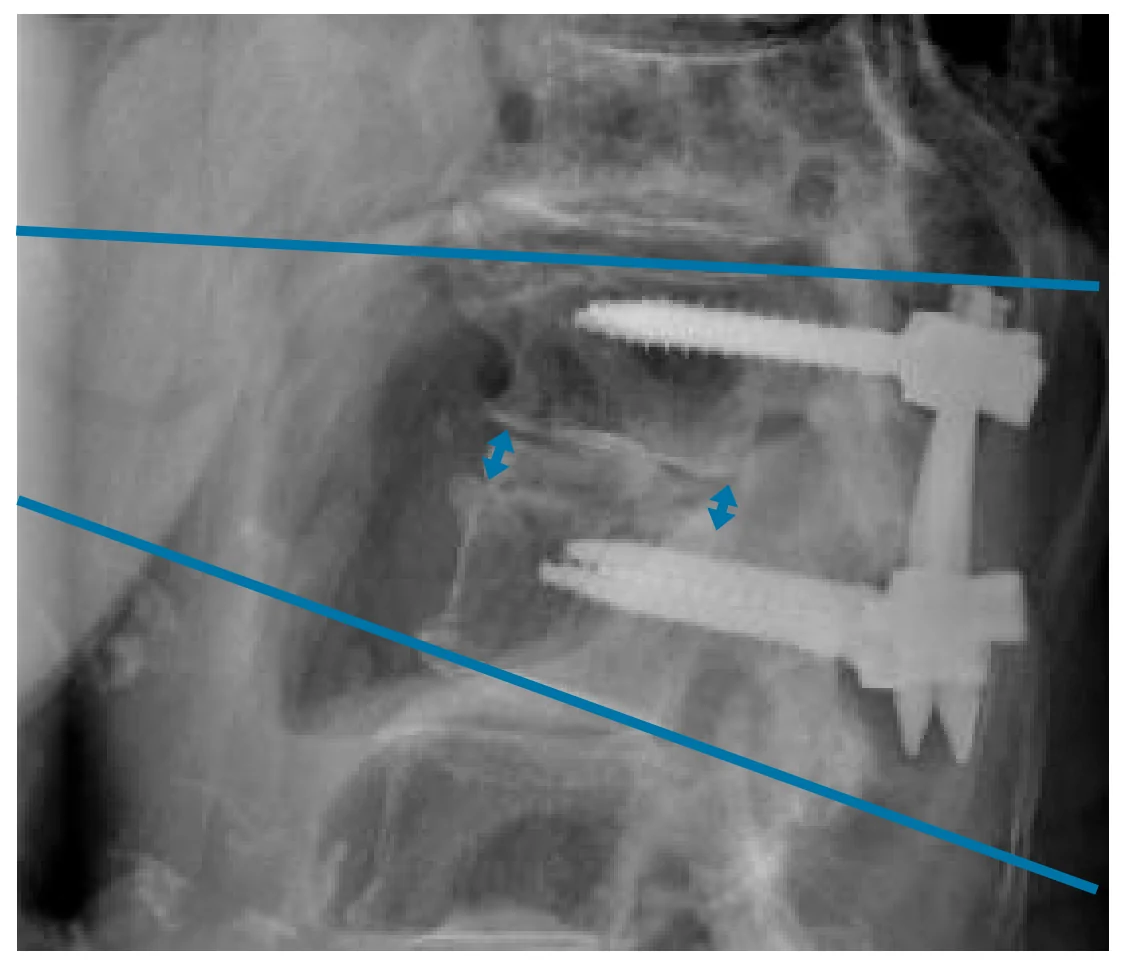
Representative sagittal imaging after mesh interbody implant implantation with posterior fixation demonstrating the measurement technique used throughout the study. The segmental Cobb angle was determined between the superior endplate of the cranial vertebra and the inferior endplate of the caudal vertebra spanning the reconstructed segment. Anterior and posterior disc heights were measured as the perpendicular distances between the opposing vertebral endplates at the anterior and posterior margins of the disc space (blue arrows).

Immediate postoperative radiographic imaging was obtained before hospital discharge according to clinical routine practice. Linear measurements were performed using the digital tools available within the institutional Picture Archiving and Communication System (PACS). No additional external calibration or correction was applied. The segmental Cobb angle was measured between the superior endplate of the cranial vertebral body and the inferior endplate of caudal vertebral body of the affected segment (Figure 1). Negative values were defined as kyphotic, positive values as lordotic. Accordingly, a positive change in Cobb angle indicates a reduction in kyphosis or shift toward lordosis. The segmental Cobb angle in multilevel reconstruction was measured across the entire reconstructed segment, from the superior endplate of the cranial vertebral body to the inferior endplate of the caudal vertebral body. Anterior and posterior disc height were measured as the perpendicular distance between the inferior endplate of the cranial vertebral body and the superior endplate of the caudal vertebral body at the anterior and posterior margins of the disc space, respectively (Figure 1). For patients undergoing multilevel cage implantation, anterior and posterior disc height were measured separately at each treated level and averaged to generate a single value per patient as an independent observation.

The different radiographic outcomes included the following: change in segmental Cobb angle and in anterior/posterior disc height (a) from baseline to immediate postoperative, (b) from immediate postoperative to 3 months, and (c) from baseline to 3 months.

Radiographic measurements were performed by a single observer. To evaluate intraobserver reliability, half of the measurements were remeasured after an interval of at least two weeks. Intraobserver agreement was assessed for segmental Cobb angle and anterior and posterior disc height using a two-way mixed-effects intraclass correlation coefficient (ICC) for absolute agreement with 95% confidence intervals [14].

### 2.5. Statistical Analysis

All statistical analyses and graphical visualizations were performed using R (Version 4.5.2). Continuous variables were summarized as median and interquartile ranges, given the small sample size and the non-normal distribution of perioperative and radiographic data. Categorical variables were reported as counts and percentages.

Radiographic changes were calculated only for patients with paired measurements at the relevant time points and were compared using the Wilcoxon signed-rank test for three comparisons:

- Baseline versus immediately postoperative;
- Immediately postoperative versus 3 months;
- Baseline versus 3 months.

All tests were two-sided and statistical significance was defined as *p* < 0.05. Because the study was exploratory and no adjustment for multiple comparisons was performed, *p*-values were interpreted descriptively. No attempt was made to standardize operative technique owing to the heterogeneity of the underlying pathologies in this cohort.

## 3. Results

### 3.1. Patient Cohort and Baseline Characteristics

Twelve patients met the inclusion criteria and had both preoperative and postoperative imaging available for analysis. The median age was 67.5 years (interquartile range [IQR], 51.2–77.9 years), and 8 patients (66.7%) were male. Median BMI was 28.3 kg/m^2^ (IQR, 23.5–32.7 kg/m^2^). Most patients were classified as ASA III (75%), while 2 patients (16.7%) were ASA II and 1 patient (8.3%) was ASA IV. Four procedures (33.3%) were revision surgeries (Table 1).

Eight reconstructions (66.7%) involved the lower thoracic spine and 4 (33.3%) involved the thoracolumbar junction. The underlying pathologies included different entities, such as trauma or fracture (25%), disc herniation, myelopathy, degenerative disease, mechanical failure or pseudarthrosis (16.7% each). One case was infection-related (8.3%). The conformable mesh interbody implant was implanted using an open Transforaminal approach in 8 cases (66.7%), an endoscopic transforaminal approach in 2 (16.7%) and a minimally invasive transforaminal approach or open posterior approach in 1 case each (8.3%). The median operative time was 226 min (IQR, 183–340 min), the median estimated blood loss was 275 mL (IQR, 78–638 mL), and the median hospital length of stay was 10 days (IQR, 6–10 days, Table 1). No new postoperative neurological deficits were identified in the available records. One patient (8.3%) developed a postoperative abscess requiring surgical washout. A second patient experienced delayed wound healing that was managed without revision surgery. No durotomies, hardware failures or other implant-related reoperations occurred during the available follow-up period. Patient-level clinical, surgical, imaging and radiographic characteristics are summarized in Supplementary Table S1.

### 3.2. Radiologic Findings

#### 3.2.1. Segmental Alignment

Median segmental Cobb angle improved from −16.2° (IQR, −21.8° to −13.8°) preoperatively to −7.9° (IQR, −9.8° to −5.9°) immediately postoperatively. At 3 months, the median angle was −7.7° (IQR, −10.6° to −5.5°). In the paired analysis, the median change from preoperative imaging to immediate postoperative examination was +6.4° (IQR, +5.0° to +11.8°; *p* = 0.003), indicating a significant reduction in segmental kyphosis. Between the immediate postoperative examination and 3-month follow-up, the median change was −1.3° (IQR, −1.5° to −1.0°; *p* = 0.067), consistent with a small degree of early correction loss that did not reach statistical significance. Compared with the preoperative measurement, the improvement remained evident at 3 months with a median change of +5.5° (IQR, +4.4° to +12.9°; *p* = 0.008, Table 2, Panel A–B).

#### 3.2.2. Anterior Disc Height

The median anterior disc height increased from 6.0 mm (IQR, 5.6–6.7 mm) preoperatively to 9.2 mm (IQR, 8.0–10.1 mm) immediately postoperatively. At 3 months, the median anterior disc height was 8.8 mm (IQR, 8.4–9.2 mm). The median paired increase from preoperative imaging to the immediate postoperative examination was 2.6 mm (IQR, 1.9–5.1 mm; *p* = 0.004). From the immediate postoperative examination to 3 months, anterior disc height decreased by a median of 0.7 mm (IQR, 0.1–1.0 mm; *p* = 0.030). Despite this early reduction, anterior disc height remained greater than baseline at 3 months, with a median preoperative-to-3-month increase of 2.3 mm (IQR, 1.7–3.3 mm; *p* = 0.013, Table 2, Panel A–B).

#### 3.2.3. Posterior Disc Height

The median posterior disc height increased from 4.3 mm (IQR, 3.4–5.7 mm) preoperatively to 7.6 mm (IQR, 5.5–8.3 mm) immediately postoperatively and measured 7.1 mm (IQR, 6.2–8.3 mm) at 3 months.

The median paired increase from preoperative imaging to the immediate postoperative examination was 2.3 mm (IQR, 0.8–3.5 mm; *p* = 0.021). Posterior disc height remained largely unchanged between the immediate postoperative examination and 3-month follow-up, with a median change of −0.2 mm (IQR, −1.1 to +0.2 mm; *p* = 0.624). Relative to the baseline, posterior disc height remained increased at 3 months by a median of 2.1 mm (IQR, 0.9–3.5 mm; *p* = 0.014, Table 2, Panel A–B).

#### 3.2.4. Individual Radiographic Trajectories

**Figure 2:**
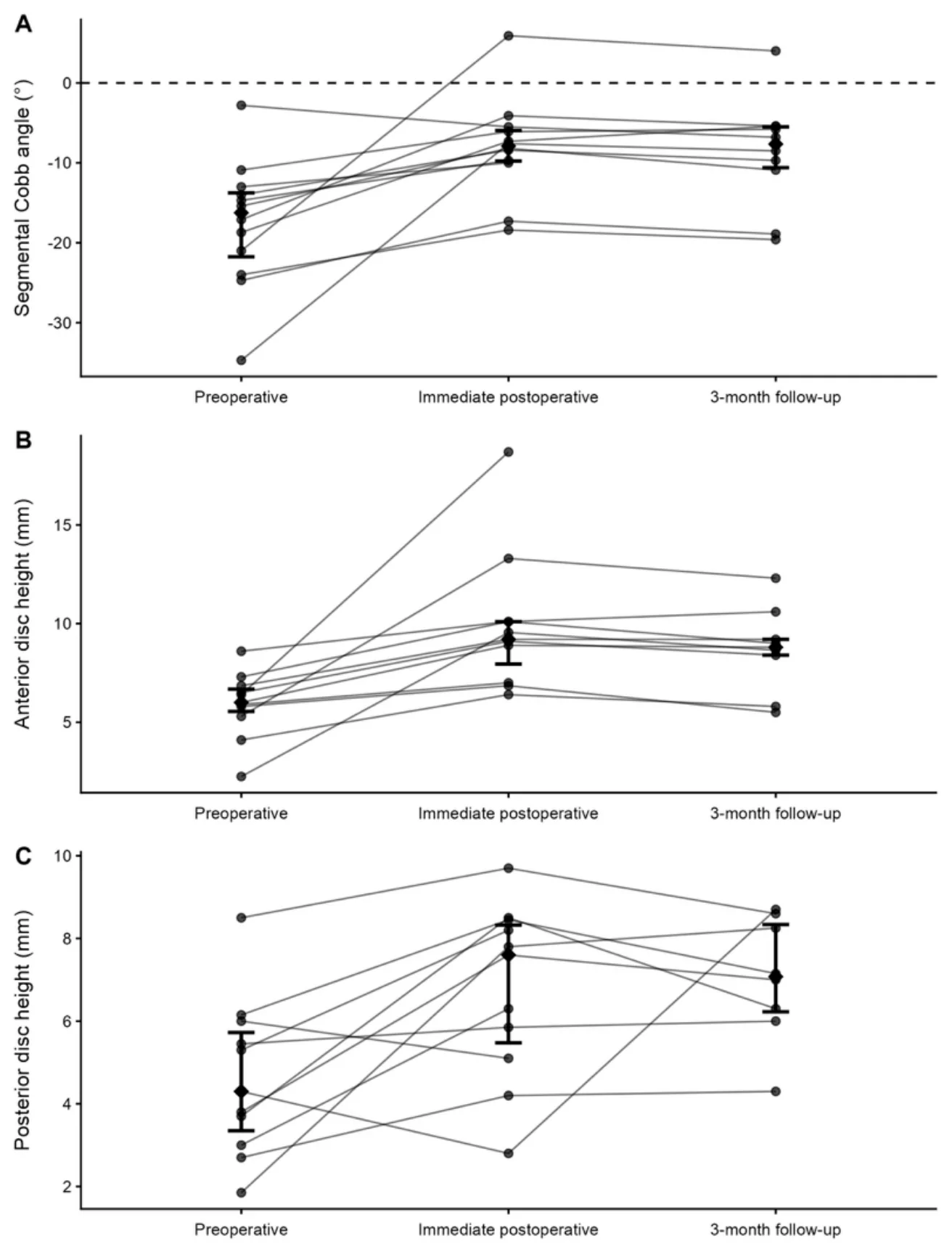
Individual radiographic outcomes. Individual changes in segmental Cobb angle (**A**), anterior disc height (**B**) and posterior disc height (**C**) from preoperative to immediate postoperative imaging and 3-month follow-up. Thin grey lines and points represent individual patients; black diamonds and vertical bars represent medians and interquartile ranges; negative Cobb angles indicate kyphosis.

**Figure 3:**
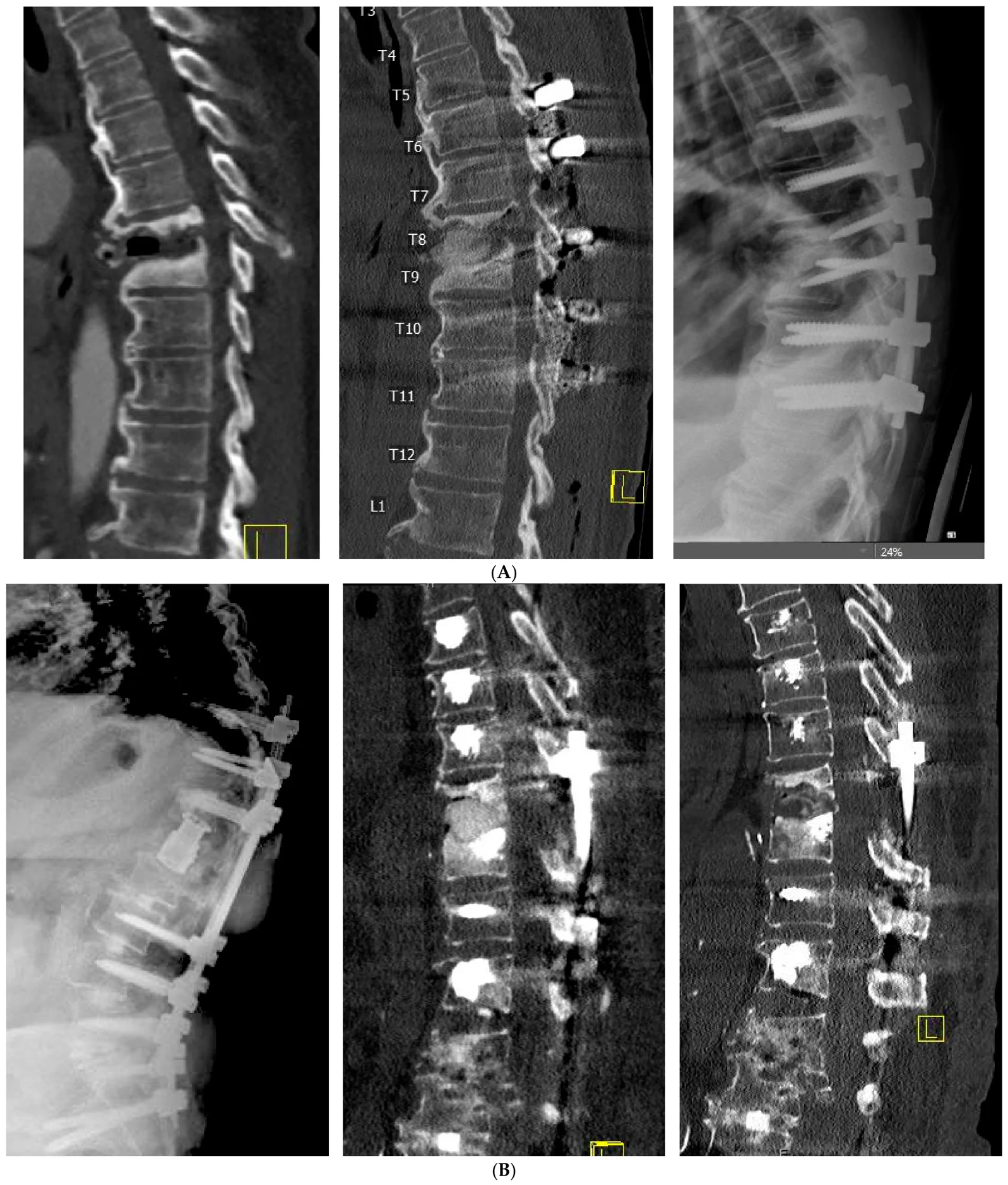

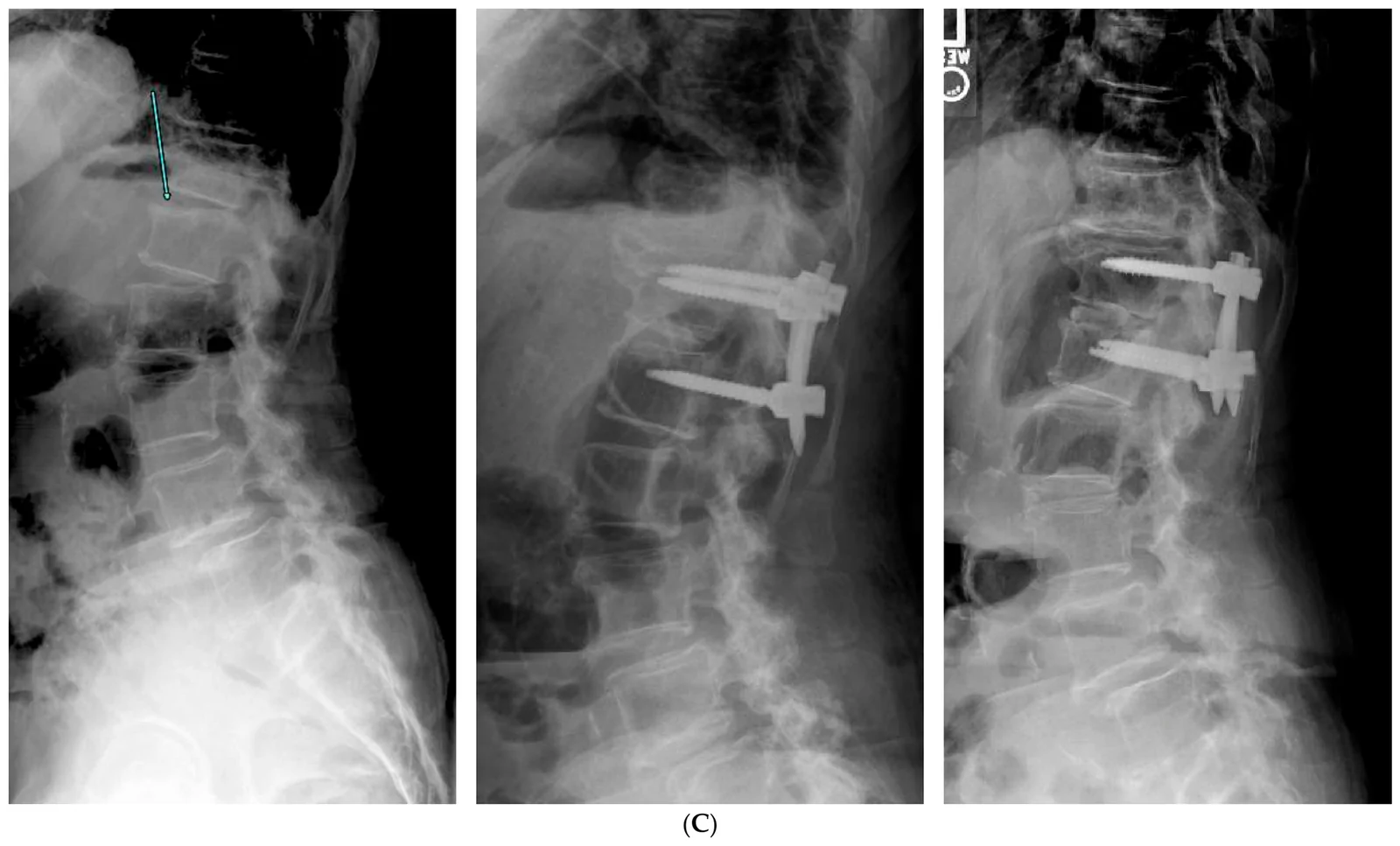
Representative cases of thoracic and thoracolumbar anterior column reconstruction using a conformable mesh interbody implant. (**A**) Case 1: This patient underwent a thoracic T8/9 reconstruction using a conformable mesh interbody implant and a posterior fixation from T6 to T11. The segmental Cobb angle improved from −17.1° at baseline to −4.1° immediately postoperatively and was maintained at −5.4° at 3 months. (**B**) Case 2: This patient underwent an open T9/10 reconstruction in the setting of a severe thoracic deformity and prior fusion and dorsal fixation approach. The segmental Cobb angle improved from −34.7° preoperatively to −7.6° immediately postoperatively and −8.5° at 3 months. (**C**) Case 3: The patient underwent an endoscopic T12/L1 reconstruction for thoracolumbar stenosis, disc herniation and myelopathy (blue arrow indicating T12/L1 level). The segmental Cobb angle in the affected segment improved from −14.0° at baseline to −8.4° immediately postoperatively and −9.7° at 3 months.

The individual longitudinal trajectories demonstrated substantial between-patient variability in the magnitude of correction and disc-height increase (Figure 2). Most patients showed an immediate postoperative reduction in segmental kyphosis together with an increase in both anterior and posterior disc height. Although some loss of the anterior disc height improvement and segmental correction of the kyphosis occurred in the early phase, the majority of available measurements remained improved at 3 months relative to baseline status. The posterior disc height was comparatively stable during the follow-up interval until 3 months. Representative cases demonstrating the range of individual trajectories from preoperative imaging through immediate postoperative assessment and 3-month follow-up are presented in Figure 3.

#### 3.2.5. Measurement Reliability

Radiographic measurements demonstrated high intraobserver reliability for the segmental Cobb angle and anterior and posterior disc height (ICC (A,1): 0.961, 95% CI: 0.896–0.986; 0.970, 95% CI: 0.913–0.990; 0.918, 95% CI: 0.773–0.973) [14].

## 4. Discussion

### 4.1. Principal Findings

This study demonstrates that thoracic and thoracolumbar reconstruction of the anterior column and sagittal alignment using a conformable mesh interbody implant through a posterior approach is feasible, even across a heterogeneous spectrum of pathologies, such as degenerative disease, trauma, infection and hardware failure. Immediate postoperative imaging demonstrated significant restoration of the segmental alignment as well as of the anterior and posterior disc height. Although a small reduction in anterior disc height and partial loss of the segmental correction were observed during the first three months of follow-up, the achieved correction remained significantly improved at 3 months compared with the baseline. The preservation of anterior column height through a posterior approach may contribute to physiological load sharing across the reconstructed segment and reduce mechanical stress on the posterior instrumentation. The posterior disc height remained largely preserved throughout the follow-up period.

Instead of demonstrating superiority over well-established reconstructive techniques, these findings suggest that anterior column support can be achieved using a conformable mesh interbody implant, particularly in challenging pathologies in which insertion of a conventional rigid interbody cage may be technically demanding.

### 4.2. Clinical Relevance

The reconstruction of the anterior column remains a technical challenge in thoracic spine surgery [4,5]. Compared with the lumbar spine, the working corridor is substantially smaller because of the presence of the spinal cord itself, pedicles, rib heads and the surrounding thoracic cage. In addition, revision surgery may be accompanied by distorted anatomy, epidural scars or pre-existing instrumentation that may further narrow the insertion channel for interbody implants.

Compromised or eroded vertebral endplates presented an additional reconstructive challenge in this cohort. Resistance to cage subsidence depends on endplate integrity, regional endplate strength, implant position, and the surface area over which load is transferred [15,16,17]. Cadaveric biomechanical studies have demonstrated that smaller foot-print cages tolerate lower loads before endplate failure and that disruption of the bony endplate substantially reduces its compressive strength [16,17]. Clinical studies of transforaminal lumbar interbody fusions have similarly associated endplate injury, a smaller cage foot print, poor bone quality, and posterior cage positioning with subsidence [18,19]. Conventional transforaminal cages are constrained by their insertion profile and provide a relatively small, fixed contact surface; effective implant–endplate contact may be further reduced when the endplates are eroded or geometrically irregular [19]. Laboratory testing suggests that in situ expansion of a conformable mesh implant may increase endplate apposition and distribute load over a broader area [20]. This proposed mechanism remains hypothetical in the present cohort because implant–endplate contact and CT-defined subsidence were not directly evaluated.

A conformable mesh interbody implant may address both the restricted access corridor and the irregular geometry of the reconstructed intervertebral space. Unlike conventional rigid or expandable cages, the implant is introduced before reaching its final dimensions and subsequently expanded by progressive in situ graft impaction [8].

Consequently, the dimensions of the approach corridor required for implantation are considerably smaller than that of a conventional cage or an expandable vertebral body implant. From a surgical perspective, this characteristic may facilitate anterior column reconstruction through a posterior or posterolateral approach while avoiding additional anterior column exposure and potentially reducing approach-related morbidity.

The presented cohort reflects precisely these clinical scenarios. One third of the patients underwent revision surgery and the indications extended well beyond isolated degenerative disc diseases to include fractures, infection, pseudarthrosis and complex reconstructions. Despite this challenging heterogeneity, radiographic restoration of alignment and disc height was consistently achieved.

### 4.3. Comparison with Previous Research

The existing clinical evidence regarding conformable mesh implants remains limited and has almost exclusively focused on lumbar degenerative disc diseases [8,10,21]. The prospective multicenter SCOUT trial demonstrated favourable outcomes, high fusion rates and a sufficient safety profile after single-level lumbar interbody fusion using a conformable interbody implant [8]. However, it intentionally evaluated a relatively homogeneous degenerative population and did not address more complex thoracic reconstructive applications with its inclusion criteria.

To our knowledge, this study represents one of the first dedicated radiographic series evaluating the feasibility of conformable mesh technology in the thoracic and thoracolumbar spine, addressing two important aspects. First, it evaluates the use of a conformable mesh implant in the thoracic and thoracolumbar spine, while published experiences remain rare. Second, it includes a broad spectrum of pathologies encountered in routine clinical spine practice rather than a highly selected degenerative cohort.

### 4.4. Interpretation of Radiographic Findings

The observed radiographic behaviour resembles the reported evidence for conformable mesh interbody implants. Immediate restoration of disc height and segmental correction was followed by a small reduction in anterior disc height during early follow-up, whereas most of the initial correction remained preserved. The clinical significance and underlying mechanism of early radiographic changes cannot be determined from the present data [22].

Several mechanisms may explain these findings. Early implant settling into the prepared vertebral endplates and graft compaction during the early postoperative period may contribute to a small reduction in anterior disc height, a phenomenon commonly observed following interbody fusion, without necessarily compromising construct stability [23,24]. Differences in imaging modality, projection or loading conditions may also contribute to these findings. However, the present data do not permit these mechanisms to be distinguished, and changes in measured disc height should not be interpreted as CT-defined implant subsidence.

These observations emphasize that the immediate postoperative radiographic appearance should not be regarded as the final result of the reconstructed segment. Despite these early radiographic changes, most of the achieved correction was maintained at 3 months. Longer-term standardized imaging is required to determine the subsequent behaviour of the reconstructed segment.

### 4.5. Limitations

Several limitations should be acknowledged. This was a retrospective single-centre study with a relatively small sample size, limiting statistical power and precluding meaningful subgroup analyses according to pathology or surgical technique. Furthermore, the cohort was intentionally heterogeneous and included different pathologies, such as trauma, infection or pseudarthrosis. Although this heterogeneity reflects the intended clinical application of the implant, it also limits direct comparison with studies focusing on a single pathology.

The follow-up duration was limited to early postoperative imaging until 3 months. Consequently, conclusions regarding fusion rates, long-term maintenance of correction or degree of implant subsidence cannot be evaluated.

The radiographic assessment was conducted by a single observer. Although intraobserver repeatability was established using repeated measurements and intraclass correlation coefficients, interobserver reliability was not evaluated. In addition, serial measurements were obtained using different imaging modalities and under non-uniform patient conditions, which may limit direct comparability across the time points, particularly for small changes. Although measurements were obtained using the PACS-based digital measurement tools, no additional external calibration or magnification correction was performed. Residual differences in projection and magnification may therefore have influenced serial linear measurements. Furthermore, the non-uniform CT follow-up precluded standardized assessment of CT-defined implant subsidence and osseous fusion. Future studies with standardized computed tomography protocols and longer follow-up are needed to address these implant-related radiographic outcomes. The observed changes in segmental alignment reflect the combined effects of the overall reconstructive procedure. The contribution of the conformable mesh implant itself cannot be isolated from the full reconstructive procedure.

Patient-reported outcome measures were not included as they were not collected consistently across the study cohort. Finally, the absence of a control group prevents comparison with conventional rigid or expandable implants. Accordingly, the presented results should be interpreted as descriptive evidence of feasibility rather than as proof of superiority.

## 5. Conclusions

Anterior column reconstruction through a posterior or posterolateral corridor using a conformable mesh interbody implant was feasible in the lower thoracic spine and thoracolumbar junction across a heterogeneous cohort of complex pathologies. The technique achieved significant early restoration of segmental alignment and anterior and posterior disc height. Most of the initial correction was maintained at 3 months, supporting short-term maintenance of radiographic correction. Larger comparative studies with longer follow-up are needed to assess fusion, progressive subsidence, and long-term construct durability.

## Figures and Tables

**Table 1:** Baseline, pathology, and operative characteristics.

	Overall Cohort (N = 12)
Demographic characteristics	
Age, years	67.5 [51.2–77.9]
Sex	
Female	4 (33.3%)
Male	8 (66.7%)
BMI, kg/m^2^	28.3 [23.5–32.7]
ASA class	
ASA 2	2 (16.7%)
ASA 3	9 (75.0%)
ASA 4	1 (8.3%)
Clinical and surgical characteristics	
Revision surgery	4 (33.3%)
Anatomical region	
Thoracic	8 (66.7%)
Thoracolumbar	4 (33.3%)
Primary pathology	
Disc herniation	2 (16.7%)
Myelopathy	2 (16.7%)
Degenerative stenosis	2 (16.7%)
Trauma/fracture	3 (25.0%)
Mechanical failure/pseudarthrosis	2 (16.7%)
Infection	1 (8.3%)
Intervention type	
Open-Transforaminal Approach	8 (66.7%)
MIS-Transforaminal Approach	1 (8.3%)
Endo-Transforaminal Approach	2 (16.7%)
Open-Posterior Approach	1 (8.3%)
Operative time, min	226 [183–340]
Estimated blood loss, mL	275 [78–638]
Hospital length of stay, days	10 [6–10]

Data are presented as median [interquartile range] or n (%), unless otherwise indicated. Percentages were calculated among patients with non-missing data for the respective variable. BMI = Body Mass Index; ASA = American Society of Anesthesiologists; MIS = Minimally Invasive Surgery.

**Table 2:** Radiographic outcomes over time. Panel A. Absolute radiographic values. Panel B. Paired radiographic changes.

**(A)**						
**Outcome**	**Preoperative**	**Immediate Postoperative**			**3-Month Follow-Up**	
Segmental Cobb angle	−16.2 [−21.8, −13.8] (n = 12)	−7.9 [−9.8, −5.9] (n = 12)			−7.7 [−10.6, −5.5] (n = 10)	
Anterior disc height, mm	6.0 [5.6, 6.7] (n = 11)	9.2 [8.0, 10.1] (n = 11)			8.8 [8.4, 9.2] (n = 9)	
Posterior disc height, mm	4.3 [3.4, 5.7] (n = 11)	7.6 [5.5, 8.3] (n = 11)			7.1 [6.2, 8.3] (n = 8)	
**(B)**						
**Outcome**	**Comparison**		**n**	**Median Change [IQR]**		***p*** **-Value**
Segmental Cobb angle	Preoperative to immediate postoperative		12	6.4 [5.0, 11.8]		0.003
	Immediate postoperative to 3 months		10	−1.3 [−1.5, −1.0]		0.067
	Preoperative to 3 months		10	5.5 [4.4, 12.9]		0.008
Anterior disc height, mm	Preoperative to immediate postoperative		11	2.6 [1.9, 5.1]		0.004
	Immediate postoperative to 3 months		9	−0.7 [−1.0, −0.1]		0.030
	Preoperative to 3 months		9	2.3 [1.7, 3.3]		0.013
Posterior disc height, mm	Preoperative to immediate postoperative		11	2.3 [0.8, 3.5]		0.021
	Immediate postoperative to 3 months		8	−0.2 [−1.1, 0.2]		0.624
	Preoperative to 3 months		8	2.1 [0.9, 3.5]		0.014

Data are presented as median [IQR]. The number of available observations is shown in parentheses in Panel A. Changes in Panel B were calculated only for patients with paired measurements. *p*-values were calculated using paired Wilcoxon signed-rank tests and are exploratory. Negative angles indicate kyphosis; therefore, a positive angular change represents reduction in kyphosis. IQR = interquartile range.

## Data Availability

The data are not publicly available due to institutional privacy regulations.

## Supplementary Materials

The following supporting information can be downloaded at https://www.mdpi.com/article/10.3390/jcm15208077/s1: Table S1: Patient-level clinical, surgical, imaging and radiographic characteristics.
