# Supplementary material for: Anterior Column Reconstruction Through a Posterior Corridor in the Lower Thoracic Spine and Thoracolumbar Junction: Early Radiographic Outcomes Using a Conformable Mesh Interbody Implant
Source: J Clin Med. 2026 Oct 17;15(20):8077. doi: 10.3390/jcm15208077 (PMC13705900; doi:10.3390/jcm15208077)
Supplement: Supplementary file 1 [file jcm-15-08077-s001.zip › jcm-4523124-supplementary.pdf]

Supplementary Table S1: Patient-level clinical, surgical, imaging and radiographic characteristics

Radiographic trajectories are reported in the order preoperative → immediate postoperative → 3-month follow-up. Imaging modality and patient position are reported for each available time point. CT and MRI were performed in the supine position. XR = X-Ray; CT = computed tomography; MRI = magnetic resonance imaging; st = standing; si = sitting; NR = not reported; NA = imaging not available; ADH = anterior disc height; PDH = posterior disc height.

Patient ID	Diagnosis	Index / Revision	Intervention Type	Posterior Fixation	Implant level(s)	Imaging Modality	Cobb Traectory (preop. - postop - 3 mos)			ADH Traectory (preop. - postop - 3 mos)			PDH Traectory (preop. - postop - 3 mos)			Complications
1	T12/L1 spinal canal stenosis	Revision	Open-Transforaminal	T7-L3	T12-L1	CT → CT → NA	-13	-10	NR	6,4	18,7	NR	3	6,3	NR	None
2	T12 burst fracture	Index	MIS-Transforaminal	T10-L2	T11-L1	XR (st) → XR (si) → XR (st)	-24,7	-17,3	-18,9	8,2/5,5	10/8,4	9,8/8,6	5,8/6,5	8,7/8,2	8,7/5,6	None
3	T12/L1 spinal canal stenosis, myelopathy	Index	Endo-Transforaminal	T12-L1	T12-L1	XR (st) → XR (st) → XR (st)	-14	-8,4	-9,7	8,6	10,1	9	3,7	8,5	6,3	None
4	T12-L2 spinal canal stenosis; L1/2 pseudoarthrosis; junctional kyphosis	Revision	Open-Transforaminal	T10-Ala ilium	T12-L2	CT → XR (st) → XR (st)	-21	5,9	4	1,7/2,8	9,1/10	8,8/8,5	2,2/1,5	10/5,6	8,2/8,3	None
5	T9/T10 spinal canal stenosis	Index	Open-Transforaminal	T7-Ala Ilium	T9-T10	XR (st) → CT → CT	-34,7	-7,6	-8,5	6	8,9	8,8	3,8	7,6	7	None
6	T12 hardware failure with screw pullout; T11/12 distraction injury	Revision	Open-Transforaminal	T8-S1	T11-T12	CT → CT → CT	-18,7	-7,3	-5,4	NR	NR	NR	NR	NR	NR	Postoperative abscess requiring surgical washout
7	T8/T9 chronic vertebral osteomyelitis	Index	Open-Transforaminal	T6-T11	T8-T9	CT → CT → XR (si)	-17,1	-4,1	-5,4	6,5	9,1	8,4	5,3	8,2	NR	Delayed wound healing, no revision surgery
8	T11/T12 disc herniation	Revision	Open-Transforaminal	T2-T12	T11-T12	MRI → CT → XR (NR)	-24	-18,4	-19,6	7,3	10,1	10,6	8,5	9,7	8,6	None

9	T11 burst fracture	Index	Endo-Transforaminal	T8-L1	T10-T11	CT → XR (si) → XR (si)	-10,9	-6,1	-5,8	5,3	13,3	12,3	4,3	2,8	8,7	None
10	T9-11 spondylosis with cord compression	Index	Open-Transforaminal	T9-T11	T9-T11	CT → XR (st) → XR (NR)	-2,8	-5,5	-6,8	6.2 / 5.4	6.4/7.3	5,9/ 5,1	4.1/ 6.8	5.5/6.2	6,0/6,0	None
11	T11/T12 spinal canal stenosis	Index	Open-Transforaminal	T10-T11	T10-T11	CT → XR (st) → XR (NR)	-15,4	-8,2	-10,9	4,1	6,4	5,8	2,7	4,2	4,3	None
12	T11/T12 disc herniation with cord compression	Index	Open-Posterior	T11-T12	T11-T12	MRI → XR (st) → NA	-14,7	-9,7	NR	5,9	7	NR	6	5,1	NR	None
